# Molecular phylogeography of East Asian *Boea clarkeana* (Gesneriaceae) in relation to habitat restriction

**DOI:** 10.1371/journal.pone.0199780

**Published:** 2018-07-03

**Authors:** Ying Wang, Kun Liu, De Bi, Shoubiao Zhou, Jianwen Shao

**Affiliations:** 1 College of Life Sciences, Anhui Normal University, Wuhu, Anhui, China; 2 Anhui Provincial Key Laboratory of the Conservation and Exploitation of Biological Resources, Wuhu, Anhui, China; 3 College of Environmental Science and Engineering, Anhui Normal University, Wuhu, Anhui, China; National Cheng Kung University, TAIWAN

## Abstract

Subfamily Cyrtandroideae (Gesneriaceae) comprises a broadly distributed group of rocky-slope herbs, with China being the center of its distributional range. The normal growth of many species within the family is particularly dependent on special habitats. Due to the paucity of molecular studies, very little is known regarding East Asian herb phylogeographic pattern. Here, we investigate the molecular phylogeography of *Boea clarkeana* Hemsl., a unique resurrection herb endemic to China, focusing on geographically restrictive effects of habitat distribution on evolutionary history. Variation in three chloroplast DNA (cpDNA) intergenic spacers (*psb*A-*trn*H, *rps*12-*rpl*20, and *trn*L-*trn*F), the ribosomal internal transcribed spacer (ITS) and simple sequence repeats in expressed sequence tags (EST-SSRs) was investigated across 18 populations to assess genetic diversity, genetic structure and historical dynamics. Genetic diversity was low within populations (cpDNA, *h*_S_ = 0.03, *π*_S_×10^3^ = 0.17; ITS, *h*_S_ = 0.16, *π*_S_×10^3^ = 0.43) but high for species (cpDNA, *h*_T_ = 0.82, *π*_T_×10^3^ = 3.12; ITS, *h*_T_ = 0.88, *π*_T_×10^3^ = 6.39); 76 alleles were detected in this highly inbred species (*F*_IS_ = 0.22), with a significantly low average of 1.34 alleles per locus. No cpDNA or ITS haplotypes were shared between regions. Based on cpDNA results, the Mt. Huangshan-Tianmu and Mt. Qinling-Daba haplotypes are ancestral; these two regions represent potential refugia. Although no evidence of significant retreat-migration phenomena during glacial cycles was detected, interglacial range expansion from northern Mt. Qinling-Daba was identified (121,457 yr BP). Rapid agricultural growth caused bottlenecks in many populations, especially on Mt. Huang-Tianmu. Habitat restriction and fragmentation, weak seed and pollen dispersal abilities, and long-term isolation caused by human-induced or environmental changes are considered the main causes of extinction of several populations and low genetic diversity within populations and regions. These analyses clarify the effects of habitat restriction on *B*. *clarkeana*, representing an evolutionary reference for similar gesneriads, and enrich our understanding of the molecular phylogeography of East Asian rocky-slope herbs.

## Introduction

A stable climate, mature plant communities in vast landscapes, high spatial heterogeneity and a long evolutionary history have laid the foundation for floristic richness in China. Accordingly, this area is an important region of diversity in the Northern Hemisphere (with a very high level of endemism [1]) and an important center for species conservation, speciation and evolution [2]. However, molecular phylogeographic studies of this area have largely concentrated on woody (mostly relic) plants [3–12]. Compared with long-lived woody plants, herbs undergo far more life cycles within a given time period and are therefore expected to respond more quickly to environmental changes; thus, herbaceous plants may provide better opportunities to study the drivers of diversification and speciation [13, 14]. However, to our knowledge, only a few herbs in this area, such as *Dysosma versipellis*, *Primula ovalifolia* and Chinese weedy rice (*Oryza stavia* L. f. sp. *spontanea*), have been studied to date [13, 15–17].

Gesneriaceae is a family of aesthetically appealing herbs with high ornamental value, among which the center of the distributional range of subfamily Cyrtandroideae occurs in China [18]. As a group of rocky-slope herbs, many species within the family have special requirements regarding soil types and habitats and are generally found in narrow areas [19]. Due to the many rare and endangered species in this family as well as a number of resurrection plants, which are extremely rare among other angiosperm groups [20], Gesneriaceae has attracted widespread attention from researchers [19, 21]. However, previous studies on Gesneriaceae have mainly focused on aspects such as population distribution, ecological protection, cytology, and ornamental value [19] or on molecular mechanisms and recovery processes (e.g., in *Boea crassifolia* and *Boea hygrometrica* [22–25]). In contrast, there have been few studies involving DNA sequences [26–28], and no research is available regarding the molecular phylogeography of these plants, even though such information may aid in understanding genetic structures and geographical distribution patterns in relation to the intrazonal distribution of Gesneriaceae.

*Boea clarkeana* Hemsl., a small perennial plant endemic to China, has remarkable light-blue flowers that confer high horticultural value [18]. *Boea clarkeana* is a typical resurrection plant of Gesneriaceae that exhibits excellent desiccation tolerance (DT) due to the ability to recover even after most of its cellular water has been lost for a period of time [20]. As a valuable gene pool for DT, *B*. *clarkeana* presents great research value, especially regarding the molecular mechanisms of DT, which may be helpful for crop cultivation [29]. Compared with other species of Gesneriaceae, *B*. *clarkeana* often shows restricted growth on rock outcrops (mostly poor limestone) in the shade of shrubs and trees [30] and has a much wider distribution area [18]. It is currently mainly distributed in China in a long, pear-shaped area along the middle-lower reaches of the Yangtze River [18, 31]. The genetic connectivity between populations of this plant mainly depends on entomophilous pollination and windborne seed dispersal. In recent years, suitable habitats have become more fragmented, and the numbers and sizes of populations have decreased significantly due to interference from human activities. Although *B*. *clarkeana* has become a subject of scientific interest in the last few years [30, 32, 33], its genetic diversity and structure, which are basic characteristics related to protection and utilization of the species, remain unknown.

Here, the population biodiversity and demographic history of *B*. *clarkeana* are examined based on variations in simple sequence repeats, or microsatellites, in expressed sequence tags (EST-SSRs) and specific regions of cpDNA (intergenic spacers, IGS) and nrDNA (entire internal transcribed spacer, ITS) in samples from 18 populations. The glacial refugia (during the last glacial maximum, LGM) and colonization routes in the postglacial (expansion) period for this plant were identified according to the results of these analyses. Overall, the results of this study enable a better understanding of the influence of habitat restriction on *B*. *clarkeana*, which represents a good evolutionary reference for similar plant species of Gesneriaceae. Furthermore, this study enriches the published research regarding the molecular phylogeography of rocky-slope herbs of East Asia.

## Materials and methods

### Sampling, experimental design and DNA extraction

**Fig 1:**
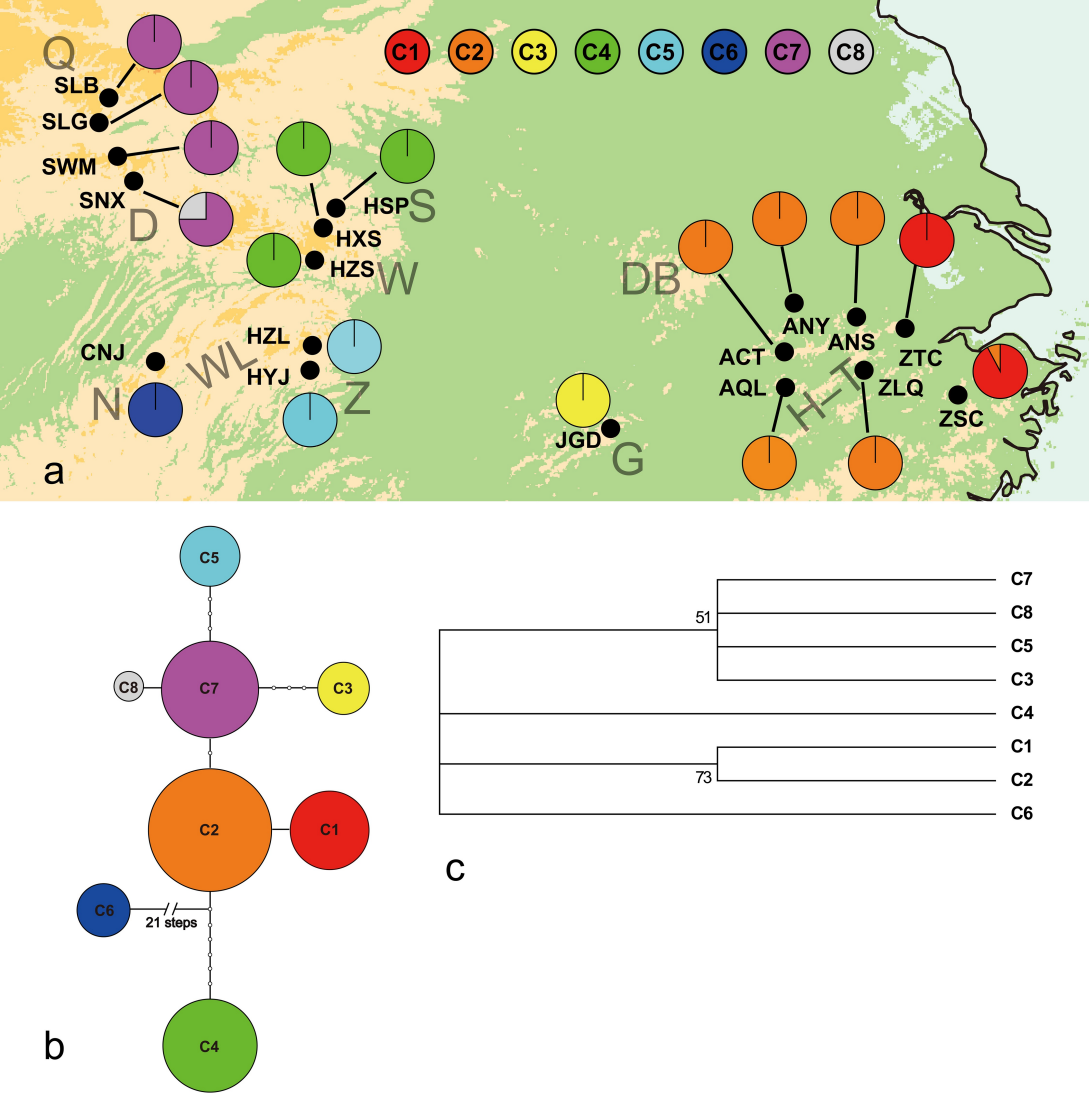
**(a) Distribution of 8 Chlorotypes of *B*. *clarkeana*.** H-T, Mt. Huang and Tianmu; G, Guan Mts.; S, Shennongjia Mts.; Z, Zhangjiajie Mts.; W, Wu Mts.; WL, Wuling Mts.; N, Nan Mts.; Q, Qinling Mts.; D, Daba Mts.; DB, Mt. Dabie. **(b) The TCS-derived Network of 8 Chlorotypes of *B*. *clarkeana*.** Small open circles indicate mutational steps. Circle sizes represent sample sizes (n). **(c) 50% Majority-Rule Consensus Neighbor-Joining Tree Obtained via Analysis of 8 Chlorotypes of *B*. *clarkeana*.** Based on 1000 permutations, bootstrap values higher than 50% are indicated above branches.

Samples were collected by and under the permit of the JiangXi Guanshan National Nature Reserve and the Protection of Wildlife Management Office of Anhui Province. A total of 394 individuals were collected from 18 natural populations covering the vast majority of the geographical range of *B*. *clarkeana* (see Table 1) [18, 31]. These samples were divided into four regional groups according to geographical distribution: Mt. Huang, Mt. Tianmu and Mt. Guan formed the eastern region; the central region included Mt. Shennongjia, Mt. Wu and Mt. Zhangjiajie; Mt. Qinling and Mt. Daba comprised the northwest region; and the southwest region included only one population (designated CNJ), located on Mt. Nan, the western-stretching branch of Mt. Wuling (see Fig 1A).

**Table 1:** Details of the samples, genetic diversity and haplotype composition of the *B*. *clarkeana* populations surveyed, based on cpDNA (*psb*A-*trn*H, *rps*12-*rpl*20, *trn*L-*trn*F), ITS and EST-SSR sequences.

Code	Location^a^	Longitude (°E)	Latitude (°N)	Size	EST-SSR^b^					cpDNA			ITS		
					*N*_A_	PIC	*H*_O_	*H*_E_	*F*_IS_	Haplotype	*h* (±SD)	π×10^3^ (±SD×10^3^)	Haplotype	*h* (±SD)	π×10^3^ (±SD×10^3^)
ZSC	T	120°79’	29°40’	21	1.533	0.1	0.093	0.118	0.373	C1,C2	0.15(±0.13)	0.08(±0.07)	R1,R2	0.15(±0.13)	0.24(±0.19)
ZTC	T	119°44’	30°32’	18	1.267	0.076	0.022	0.096	0.779	C1	0	0	R3	0	0
ZLQ	T	118°90’	30°12’	24	1.267	0.067	0.056	0.082	0.338	C2	0	0	R2	0	0
ANS	H	118°88’	30°68’	22	1.667	0.085	0.025	0.093	0.744	C2	0	0	R3	0	0
ANY	H	118°13’	30°89’	24	1.333	0.092	0.125	0.117	-0.051	C2	0	0	R1,R2,R4	0.39(±0.15)	0.78(±0.33)
ACT	H	117°63’	30°34’	24	1.533	0.095	0.111	0.111	0.017	C2	0	0	R1	0	0
AQL	H	117°49’	29°88’	24	1.4	0.088	0.106	0.105	0.012	C2	0	0	R1	0	0
JGD	G	114°56’	28°54’	24	1.133	0.008	0.008	0.008	-0.015	C3	0	0	R5	0	0
HSP	S	110°85’	31°75’	20	1.267	0.079	0.074	0.09	-0.043	C4	0	0	R6,R7,R8	0.67(±0.08)	1.51(±0.20)
HXS	S	110°64’	31°34’	14	1.007	0.005	0.005	0.005	0	C4	0	0	R8	0	0
HZS	W	110°40’	30°84’	24	1.4	0.068	0.036	0.082	0.607	C4	0	0	R8	0	0
HZL	Z	110°45’	29°09’	24	1.333	0.079	0.089	0.096	0.101	C5	0	0	R9,R10	0.57(±0.12)	3.54(±0.74)
HYJ	Z	110°44’	28°78’	24	2.333	0.27	0.278	0.309	0.121	C5	0	0	R11	0	0
CNJ	N	107°20’	29°10’	24	1.333	0.037	0.014	0.043	0.688	C6	0	0	R12	0	0
SNX	D	106°93’	32°44’	24	1.13	0.024	0.023	0.028	0.193	C7,C8	0.41(±0.13)	0.22(±0.13)	R13,R14	0.53(±0.08)	1.64(±0.24)
SWM	D	106°54’	32°57’	20	1.067	0.003	0.003	0.003	0	C7	0	0	R13	0	0
SLB	Q	106°08’	33°58’	24	1.133	0.023	0.029	0.027	-0.048	C7	0	0	R13	0	0
SLG	Q	105°94’	33°30’	15	1	0	0	0	NA	C7	0	0	R13	0	0
AVERAGE					1.34	0.07	0.06	0.08	0.22		0.03	0.17		0.16	0.43
TOTAL				394						8	0.82(±0.01)	3.12(±0.13)	14	0.88(±0.01)	6.39(±0.30)

Notes

^a^: H, Huang Mts.; T, Tianmu Mts.; G, Guan Mts.; W, Wu Mts.; S, Shennongjia Mts.; Z, Zhangjiajie Mts.; N, Nan Mts.; Q, Qinling Mts.; D, Daba Mts.

^b^, *N*_A_, number of alleles per locus across all populations; PIC, polymorphic information content; *H*_O_, observed heterozygosity (mean value); *H*_E_, expected heterozygosity (mean value); *F*_IS_, inbreeding coefficient; *h* haplotype diversity; *π*, nucleotide diversity; SD, standard deviation; NA, not available.

Leaf material was collected and dried rapidly in silicagel. All individuals (n = 394) were surveyed for population structure and genetic diversity with respect to EST-SSR variation, and cpDNA (intergenic spacer, IGS) and nrDNA (entire internal transcribed spacer, ITS) regions were sequenced in a subset of samples (n = 222). Total genomic DNA was isolated from dried leaves using QIAGEN DNeasy^®^ Plant Mini Kit (QIAGEN, Germany) following the manufacturer’s manual.

### Amplification, sequencing and microsatellite genotyping

Polymerase chain reaction (PCR) amplification was performed using a 5828R PCR instrument (MyCycler Thermal Cycler, BIO-RAD, USA) in a 15-μL volume containing 50 ng DNA, 1×PCR buffer, 2.5 mM deoxyribonucleotide triphosphates (dNTPs) and MgCl_2_, 0.5 U *Taq* polymerase (TaKaRa, Dalian, China), and 0.04 μM forward and reverse primers. The PCR conditions were as follows: initial denaturation at 94°C for 5 min, followed by 35 cycles of 30 s at 94°C, 40 s at 54°C, and 45 s at 72°C, with an additional extension at 72°C for 10 min. The PCR products were detected using an ABI 3730XL DNA Analyzer (Applied Biosystems, Foster City, USA), and alleles were scored manually using GeneMaker (version 2.2.0).

For cpDNA, six IGS regions (*psb*A-*trn*H, *rps*12-*rpl*20, *trn*L-*trn*F, *trn*S-*trn*G, *trn*T-*trn*F and *psb*B-*psb*F) were sequenced and detected in a preliminary screen [34, 35], after which three regions (*psb*A-*trn*H, *rps*12-*rpl*20 and *trn*L-*trn*F) showing stable amplification and high levels of variation were chosen for further analyses. Sequence alignments were generated using MEGA 6.0 [36]. Seventeen EST-SSR markers (BC1-BC17) that were specifically developed for *B*. *clarkeana* were used to detect genetic variation [32]. In the survey of EST-SSR variation, fluorescent M13 primers were used to accurately screen variation among individuals. All haplotype cpDNA and ITS sequences have been deposited in the GenBank database (*psb*A-*trn*H, BankIt1898693, KU867796-KU867801; *rps*12-*rpl*20, BankIt1898703, KU867802-KU867805; *trn*L-*trn*F, BankIt1898706, KU867806-KU867811; ITS, BankIt1898665, KU867781-KU867795).

### Genetic diversity and structural analyses

The genetic diversity of the cpDNA and ITS sequences was estimated based on the number of haplotypes, haplotype diversity and nucleotide diversity for the species (*h*_T_, *π*_T_) and populations (*h*_S_, *π*_S_), which were determined using DnaSP 5.1 [37]. Phylogeographic structure was evaluated using the differentiation indices *N*_ST_ (ordered haplotypes) and *G*_ST_ (unordered haplotypes), which were calculated using Permut 1.0 (1000 permutations) [38]. To detect the correlation between genetic and geographical distance and estimate genetic variance components, the Mantel test and analysis of molecular variance (AMOVA) were implemented in Arlequin 3.0 [39, 40]. Neighbor-joining (NJ) phylogenetic trees of 8 chlorotypes and 14 ribotypes of *B*. *clarkeana* were built using Mega 6.0 [36].

For EST-SSRs, Hardy-Weinberg equilibrium (HWE) was detected using the online tool GENEPOP (http://www.genepop.curtin.edu.au/), and Bonferroni’s correction was applied. We then utilized LOSITAN [41] for neutral detection of markers. Finally, 15 pairs of primers showing the greatest numbers of polymorphic loci, good amplification and agreement with the neutral theory (BC2 and BC14 did not agree with the neutral theory) were selected for the genotyping of all samples. The genetic variation of EST-SSRs was estimated based on the number of alleles (*N*_A_), observed heterozygosity (*H*_O_), expected heterozygosity (*H*_E_) and polymorphism information content (PIC) at each locus, which were calculated using GenAlEx v.6.501 [42] and PowerMarker (version 3.25) [43]. The within-population inbreeding coefficient (*F*_IS_) and among-population coefficient (*F*_ST_) were determined with FSTAT [44], and AMOVA was implemented in Arlequin 3.0 [39]. To reveal population structure based on the genetic distance among all sampled individuals, principal component analysis (PCoA) was performed with GenAlEx v.6.501 [42]. Based on the pairwise genetic distance (*D*_A_) calculated for the populations [45], an UPGMA tree was built using the program DISPAN [46].

### Contemporary and historical gene flow

The programs BayesAss (version 1.3) and Migrate (version 3.6.5) were used to estimate contemporary (over the past few generations) and historical gene flow (over a much longer period extending back approximately 4*N*_e_ generations), respectively [47, 48]. Based on the Bayesian method, population-specific *F*_IS_ and genotypic disequilibrium were detected through Markov Chain Monte Carlo (MCMC) analysis, and contemporary gene flow was measured. Each MCMC run was performed with a burn-in of 1×10^6^ and a total of 2×10^6^ iterations. Five replicates for each run were performed with different initial seeds. Delta values were adjusted to maximize log likelihood values and ensure a sufficient search of the parameter space [47]. The model convergence posterior distribution was tested using Tracer v. 1.4.8 [49] to determine whether each run displayed the best model fit. The mutation-scaled migration rate (*M*) was estimated using Migrate based on the coalescent theory. The following equation was utilized: *M* = *m*_h_ */μ*, where *μ* is the mutation rate per generation (3×10^−4^) [50], and *m*_h_ is the historical migration rate. We ran three replicates with the allele model, and the static heating scheme was applied at four temperatures (1, 1.5, 3, and 1,000,000).

### Population demographic changes

For cpDNA, mismatch distribution analysis (MDA) was performed with Arlequin 3.0 [39], which was used to evaluate the evidence of spatial and demographic expansion. For each MDA model, parametric bootstrapping was used to test the goodness of fit based on the sum of squared deviations (*SSD*) and Harpending’s raggedness index (*H*_*Rag*_) [51]. In addition, Tajima’s D [52] and Fu’s *F*_S_ [53] tests of selective neutrality were performed to assess potential spatial and demographic expansion. To estimate the time (T, in number of generations) of population expansion, the expansion parameter (*τ*), which is also derived from MDA, and the neutral mutation rate (*u*) for the entire sequence per generation were used in the equation T = *τ*/2*u* [51, 54]. The *u* value was calculated as *u* = *μkg*, where *μ* is the substitution rate per site per year (s/s/y, here 1.3×10^−9^) [15, 55], *k* is the average sequence length of the cpDNA region in the present study, and *g* is the generation time in years. The value for *k* in our study was 1900 bp, and 5 years was used as an approximation of the generation time, *g*. As no reference was available to determine generation time, we used the formula *T* = *α*+ [*S*/(1-*S*)] to calculate the generation time *T* (g), where *α* is the maturation age and *S* is the survival rate [56]. Based on observations, we set the maturation age and seed survival rate to 3 years and 60%, respectively (Professor Shoubiao Zhou, Anhui Normal University, personal communication). Therefore, a generation time (*T*) of approximately 5 years was used in this study. TCS 1.21 [57] was employed to construct haplotype networks with the connection limit set to 95%.

Based on EST-SSRs, recent bottlenecks (past 2*N*_e_-4*N*_e_ generations) and long-term demographic changes were detected with BOTTLENECK v. 1.2.02 and MSVAR v 1.3 [58, 59]. In the present study, two complementary methods were applied to detect genetic bottlenecks using the program BOTTLENECK. First, a mode-shift test was employed to detect the shifted model characteristics of bottlenecked populations. For non-bottlenecked populations, a large proportion of alleles were present at a low frequency, and the allele frequency curve followed a normal L-shaped distribution. When there are fewer (<10%) alleles with a low frequency than alleles with an intermediate frequency in bottlenecked populations, the normal L-shaped frequency distribution curve will shift [60]. Second, we used Wilcoxon's sign rank test. Three mutation models (infinite allele model, IAM; two-phase mutation model, TPM; stepwise mutation model, SSM) were fitted to detect excessive heterozygosity, and a *P*-value < 0.05 using the Wilcoxon signed rank test was considered to represent evidence of a bottleneck. MSVAR was used to detect long-term changes in the effective population size and the timing of the changes. The lineal model was employed to evaluate population size changes [61], recording every 90,000 steps, with 30,000 times recorded in total. The posterior distributions of four parameters of population dynamics (ancestral effective size (*N*_0_), current effective population size (*N*_1_), average mutation rate across loci (*μ*), and time (years) since the onset of decline or expansion (*T*)) were estimated using Tracer v. 1.4.8 [49].

## Results

### CpDNA diversity and phylogeography

By concatenating the three aligned IGS regions (*psb*A-*trn*H, 325 bp; *rps*12-*rpl*20, 739 bp; *trn*L-*trn*F, 836 bp), we obtained 1900 bp of cpDNA sequence from 222 individuals. A total of 21 sites with insertion-deletions (indels) and 35 polymorphic sites (substitutions) were observed (see S1 Table). Mutations were found to be largely distributed in *psb*A-*trn*H (22 sites), mainly consisting of sequence differences between CNJ and the other populations. Only substitutions were used in the ensuing data analysis, whereas indels were excluded. In total, 8 chlorotypes (haplotypes C1-C8) were identified (see Table 1, Fig 1). In addition, multiple chlorotypes were found for only two populations (ZSC, SNX); the other 16 populations were monomorphic. Furthermore, no chlorotypes were shared among regions, even though shared chlorotypes were common among the populations in each region. Accordingly, the species exhibited high haplotype diversity and nucleotide diversity (*h*_T_ = 0.82, *π*_T_×10^3^ = 3.12) at the species level but low diversity within each population (*h*_S_ = 0.03, *π*_S_×10^3^ = 0.17). Only the eastern (Huang, Tianmu and Guan Mts.) and northwest (Qinling and Daba Mts.) regions displayed haplotype and nucleotide diversity (*h* = 0.02, *π*_S_×10^3^ = 0.01; *h* = 0.10, *π*_S_×10^3^ = 0.06), and the other two regions (central and northwest regions) showed no diversity (*h* = 0, *π*_S_×10^3^ = 0) (see details in S2 Table). Genetic structure was further revealed by AMOVA (see Table 2); most of the variation existed among groups (95.89%, *Φ*_ST_ = 0.994), and significant phylogeographic structure was found (*N*_ST_ = 0.995 > *G*_ST_ = 0.964, both *P* < 0.05). A moderately strong relationship between the genetic and geographic distances between populations was revealed by Mantel tests (*r*_M_ = 0.54, *P* < 0.01). In the unrooted network (see Fig 1B), chlorotypes C2 (from Mt. Huang-Tianmu) and C7 (from Mt. Qinling-Daba) were located in the interior nodes (ancestral chlorotypes) of the network and differed by only two mutational steps, despite the great physical distances involved. In contrast, chlorotypes C4 and C5, from the adjacent Mt. Shennongjia-Wu and Mt. Zhangjiajie regions, differed by 13 mutational steps, indicating that these two regions were isolated and that their populations had evolved independently for a long time. Furthermore, chlorotypes C4 and C6 (from population CNJ, Mt. Nan) were distant from the other chlorotypes, especially C6, which exhibited more than 22 mutational steps differing from the other chlorotypes, indicating that it had been isolated from the other chlorotypes and had evolved into a distinct type over time. Chlorotypes C1, C3, C5, and C8 were subclades (tip chlorotypes) associated with C2 and C7, but separated from them by one to four mutational steps. The NJ tree (see Fig 1C) showed a similar structure to the network. Chlorotypes C6 and C4 each formed a distinct branch that was far from the other 6 chlorotypes. Chlorotypes C3 and C5 and chlorotypes from Mt. Qinling-Daba (C7, C8), which showed a close relationship, formed a large clade.

**Table 2:** Variation percentage and expansion tests based on cpDNA sequences of *B*. *clarkeana*.

Groups^a^	Size	Percentage of variation				*G*_*ST*_(*se*)	*N*_*ST*_(*se*)	Expansion parameter (*τ*)^b^	Expansion Time (t)	*SSD* *(P)*	*H*_*Rag*_ (*P*)	Tajima’s D (*P*)	Fu’s *F*_S_ (*P*)
		Among groups	Among populations	Within populations	*Φ*_*ST*_								
H-T	89	19.57	75.94	4.49	0.955	0.952 (0.036)	0.952 (0.036)	0 (0–0.016)	-	0.334 (0)	0.684 (0.940)	1.254 (0.913)	3.709 (0.923)
H-T-G	101	89.98	9.59	0.43	0.996	0.968 (0.029)	0.986 (0.015)	0 (0–0.646)	-	0.406 (0)	0.459 (0.930)	0.242 (0.649)	8.022 (0.984)
Z-W-S	60	100	0	0	1	1(NC)	1(NC)	3 (0–3.682)	-	0.261 (0.010)	0.701 (0.220)	2.851 (0.998)	21.511 (1.0)
WL (Z, N)	31	100	0	0	1	1(NC)	1(NC)	0 (0–0)	-	0.481 (0)	0.741 (1.0)	3.405 (1.0)	30.223 (1.0)
W-S-Q-D	90	98.56	0.29	1.15	0.989	0.904 (0.095)	0.986 (0.015)	0 (0–0.281)	-	0.511 (0)	0.663 (0.92)	3.247 (0.999)	20.055 (0.999)
Q-D	49	-1.26	19.4	81.86	0.181	0.182 (NC)	0.182 (NC)	3 (0.418–3.00)	121 457 (16 923–121 457)	0.02 (0.040)	0.807 (0.840)	-0.644 (0.238)	1.226 (0.581)
Total	222	95.89	3.49	0.62	0.994	0.964 (0.027)	0.995 (0.004)	39.324 (0–210.824)	-	0.061 (0.210)	0.124 (0.010)	0.029 (0.600)	22.282 (0.998)

Notes

^a^: See Table 1 for mountain codes.

^b^: 97.5% CI. *N*_ST_, differentiation index of ordered haplotypes; *G*_ST_, differentiation index of unordered haplotypes; *SSD*, the sum of squared deviations; *HRag*, Harpending’s raggedness index.

NC, not calculated.

### ITS diversity and phylogeography

**Fig 2:**
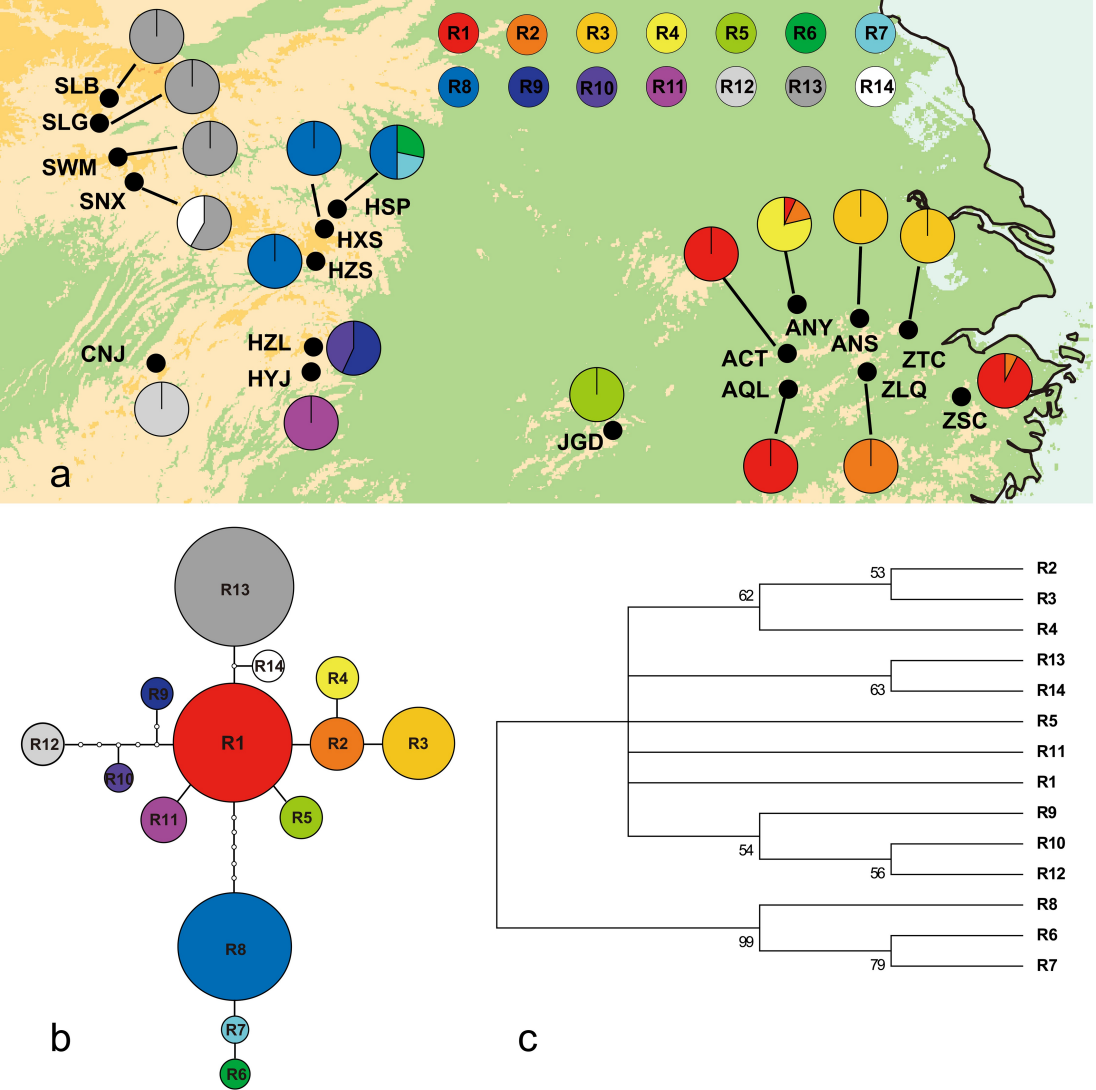
**(a) Distribution of 14 Ribotypes in *B*. *clarkeana*. (b) TCS-derived Network of 14 Ribotypes of *B*. *clarkeana***. Small open circles indicate mutational steps. Circle sizes represent sample sizes (n). **(c) 50% Majority-Rule Consensus Neighbor-Joining Tree Obtained via the Analysis of 14 Ribotypes of *B*. *clarkeana*.** Based on 1000 permutations, bootstrap values higher than 50% are indicated above branches.

ITS region sequences were aligned with a length of 663 bp. In total, 18 sites contained indels, and 22 showed nucleotide substitutions (see S3 Table). Only these substitutions were employed as polymorphic sites in the following data analysis; indels were excluded. Altogether, 14 ribotypes (haplotypes, R1-R14) were revealed by these polymorphic sites. Similar to the results of the cpDNA analysis, no ribotypes were shared between regions, only between populations within a single region (see Table 1, Fig 2A). These findings demonstrate that different populations within a region have commonly experienced genetic exchange of cpDNA and ITS sequences. Five populations (ZSC, ANY, HSP, HZL, SNX) contained multiple ribotypes, whereas the other 13 populations contained one fixed ribotype. For the populations with multiple ribotypes, population haplotype diversity and species nucleotide diversity (*h*_T_ = 0.88, *π*_T_×10^3^ = 6.39) in the ITS region were higher than in cpDNA. Among regions, the eastern and central regions showed more fixed ribotypes compared with the results for cpDNA. However, in contrast to the cpDNA results, the central region showed the highest haplotype diversity and nucleotide diversity (*h* = 0.25, *π*×10^3^ = 1.01) among the regions (see S2 Table). Similar to the cpDNA results, the southwest region exhibited no diversity in ITS sequences, largely because only one population (CNJ) was sampled in this region. Hierarchical AMOVA revealed 75.08% (*Φ*_ST_ = 0.945) variation between groups, indicating obvious phylogeographic structure (*N*_ST_ = 0.931 > *G*_ST_ = 0.862, *P* = 0.059/0.037) (see S4 Table). In contrast to the network of cpDNA chlorotypes, ribotype R1 (Mt. Huang-Tianmu) was the sole interior ribotype of the ITS network, and most ribotypes were clustered in their regions (see Fig 2B). Excluding ribotypes R6, R7, R8 (Mt. Shennongjia-Wu) and R12 (Mt. Nan), which differed from R1 by 8, 7, 6 and 6 steps, the other 10 ribotypes formed subclades (tip ribotypes) differing from R1 by one to four mutational steps. Consistent with the cpDNA results, R13 and R14 (Mt. Qinling-Daba) differed from R1 by only 2 and 1 mutational steps (Mt. Huang-Tianmu), respectively, indicating a close genetic relationship between these two regions. In contrast, the ribotypes of the Mt. Shennongjia-Wu (R6, R7 and R8) and Mt. Zhangjiajie (R9, R10 and R11) regions differed by more than 7 mutational steps, indicating long-standing isolation between these two regions. The NJ tree showed a similar structure to the ITS network (see details in Fig 2C). As a result, R6, R7 and R8 (Mt. Shennongjia-Wu) were grouped into one clade, which was distant from another large clade that contained the remaining 11 ribotypes. In contrast to the cpDNA results, the CNJ population did not comprise the branch with the greatest genetic distance in this analysis.

### Genetic diversity and structure based on EST-SSRs

**Fig 3:**
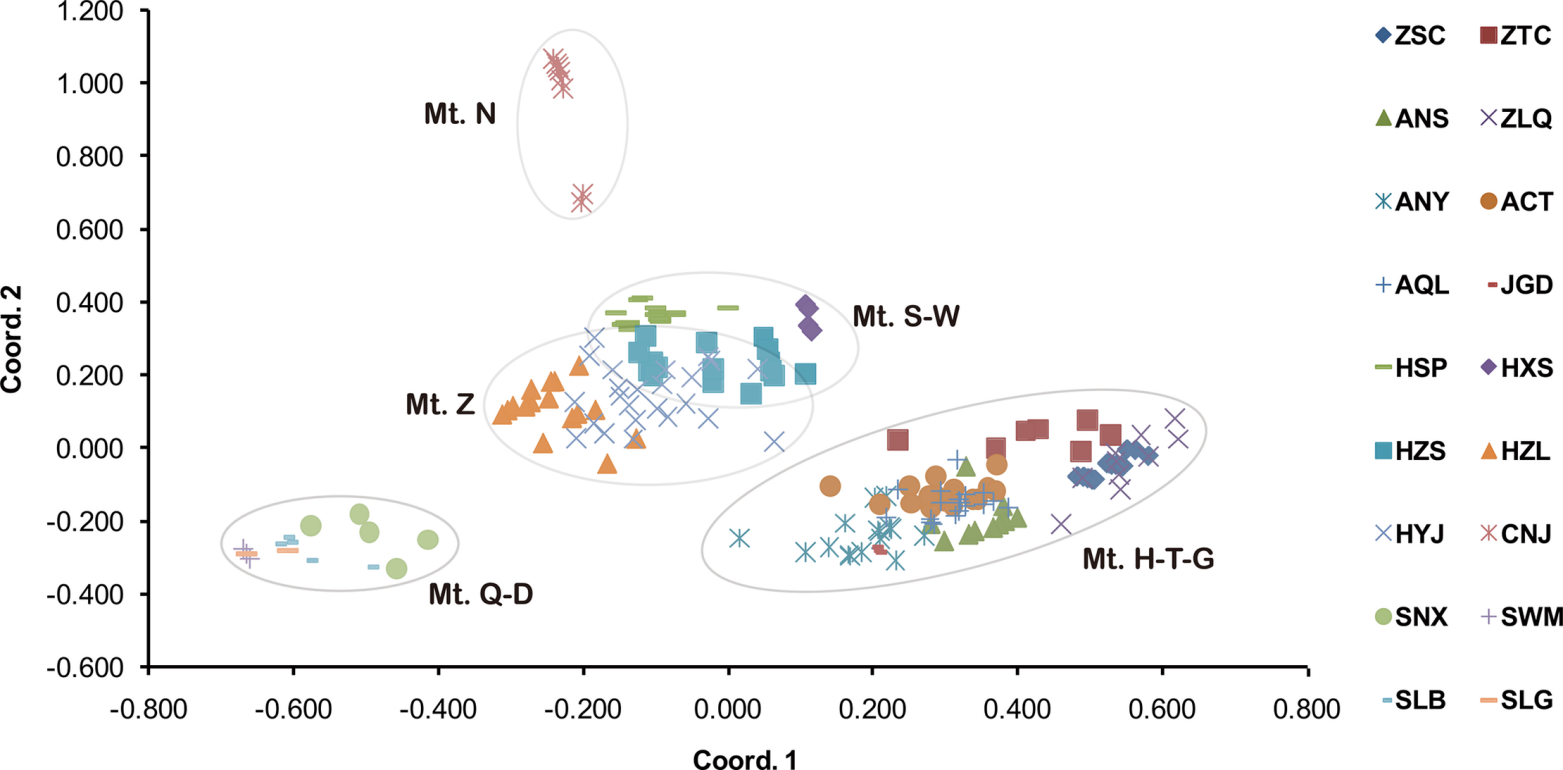
PCoA of 18 populations of *B*. *clarkeana*. See Table 1 for mountain codes.

**Fig 4:**
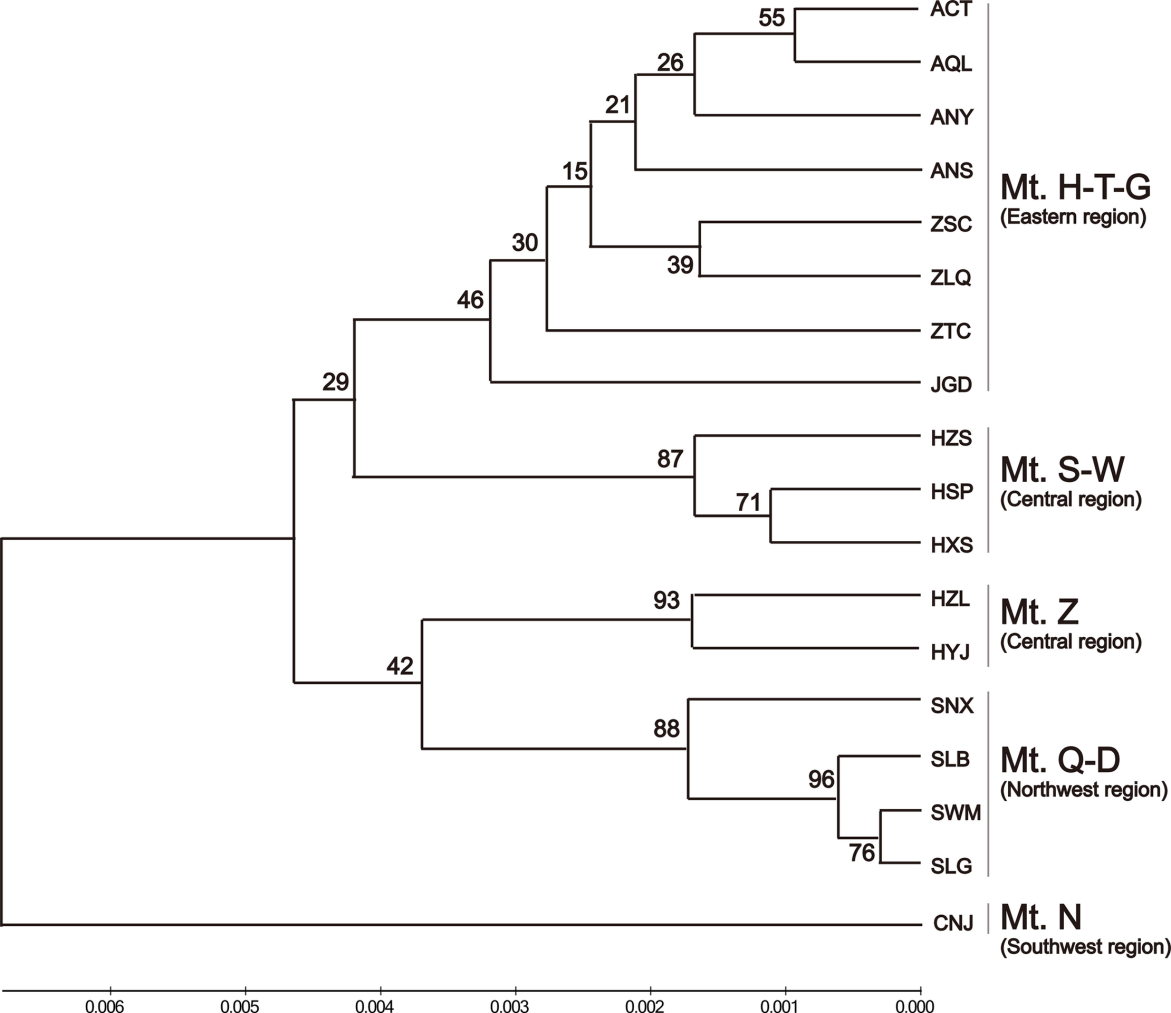
UPGMA dendrogram depicting Nei’s (1978) genetic distances (*D*_A_) between 18 populations of *B*. *clarkeana*, based on 15 EST-SSR Loci [45]. Numbers above branches indicate bootstrap values. See Table 1 for population and mountain codes.

Among the 394 individuals of *B*. *clarkeana* surveyed at 15 EST-SSR loci, 76 alleles were obtained, ranging from 2 to 9 per locus. The mean *N*_A_, PIC, and *H*_O_ and *H*_E_ values were 1.34, 0.07, 0.06 and 0.08 (see Table 1), respectively, indicating very low genetic diversity in these populations. Similar to the diversity results obtained using ITS data, the central region (Wu, Shennongjia and Zhangjiajie Mts.) exhibited the highest diversity values (*N*_A_ = 1.468, PIC = 0.1, *H*_O_ = 0.096, and *H*_E_ = 0.116) among all regions, followed by the eastern region (*N*_A_ = 1.392, PIC = 0.076, *H*_O_ = 0.068, and *H*_E_ = 0.091). The northwest region (Qinling and Daba Mts.) presented the lowest diversity (*N*_A_ = 1.08, PIC = 0.013, *H*_O_ = 0.014, and *H*_E_ = 0.015) among the regions (see S2 Table for details). In addition, most of the variation existed between groups (55.23%, *Φ*_ST_ = 0.844), and the variation between populations within groups was higher (29.19%) than the values obtained for cpDNA and ITS. The inbreeding coefficient (*F*_IS_ = 0.22) was significantly positive in 11 populations, with only 4 populations (ANY, JGD, HSP and SLB) presenting a negative value, indicating common inbreeding between populations of *B*. *clarkeana*. The genetic differentiation between populations and regions was relatively high, and most pairwise *F*_ST_ values for the 18 populations were significantly greater than 0.5, except for those among the 3 populations of Mt. Qinling-Daba (SWM and SLB, 0.199; SWM and SLG, 0.007; SLG and SLB, 0.041). Additionally, the population CNJ exhibited the highest mean pairwise *F*_ST_ value (0.916). According to PCoA, all populations grouped into their regions, which were divided into four clusters (Mt. Huang-Tianmu-Guan, Mt. Shennongjia-Wu-Zhangjiajie, Mt. Nan and Mt. Qinling-Daba). These four regions were arranged in accordance with longitude along coordinate1 (see Fig 3). Similar to the results of PCoA, all populations in the UPGMA tree grouped into geographical regions (see Fig 4), and the population relationships showed a correlation with geographic distance. The populations of the eastern region displayed great genetic distance from the populations of the southwestern or northwestern region.

### Gene flow and historical demography dynamics

Based on the EST-SSR data, contemporary and historical gene flow was detected for *B*. *clarkeana*. Low levels of contemporary gene flow were determined through multiple runs of BayesAss. In addition, contemporary gene flow between populations was found to be extremely weak, as the contemporary migration rate (*m*_c_) values were all less than 0.022. The historical migration rates (*m*_h_) calculated by Migrate ranged from 0.008 (from CNJ to SWM) to 0.248 (from SLG to SLB) (see S5 Table), with a mean value of 0.113.

Based on the results of cpDNA analysis, a unimodal mismatch distribution with non-significant *SSD* and *H*_*Rag*_ values was fitted to support the expected expansion of the distribution. In this study, Mt. Qinling-Daba was the only region satisfying the following criteria: non-significant *SSD* and *H*_*Rag*_ values (0.02, *P* = 0.04; 0.807, *P* = 0.84) and a significantly negative Tajima’s D (-0.644, *P* = 0.238). Based on the *τ*-value (3), the expansion of the Mt. Qinling-Daba region was dated to approx. 121,457 yr BP (see Table 2, S1 Fig). Other than the regional expansion observed at Mt. Qinling-Daba, no major expansion or contraction of the population size of *B*. *clarkeana* was detected.

**Fig 5:**
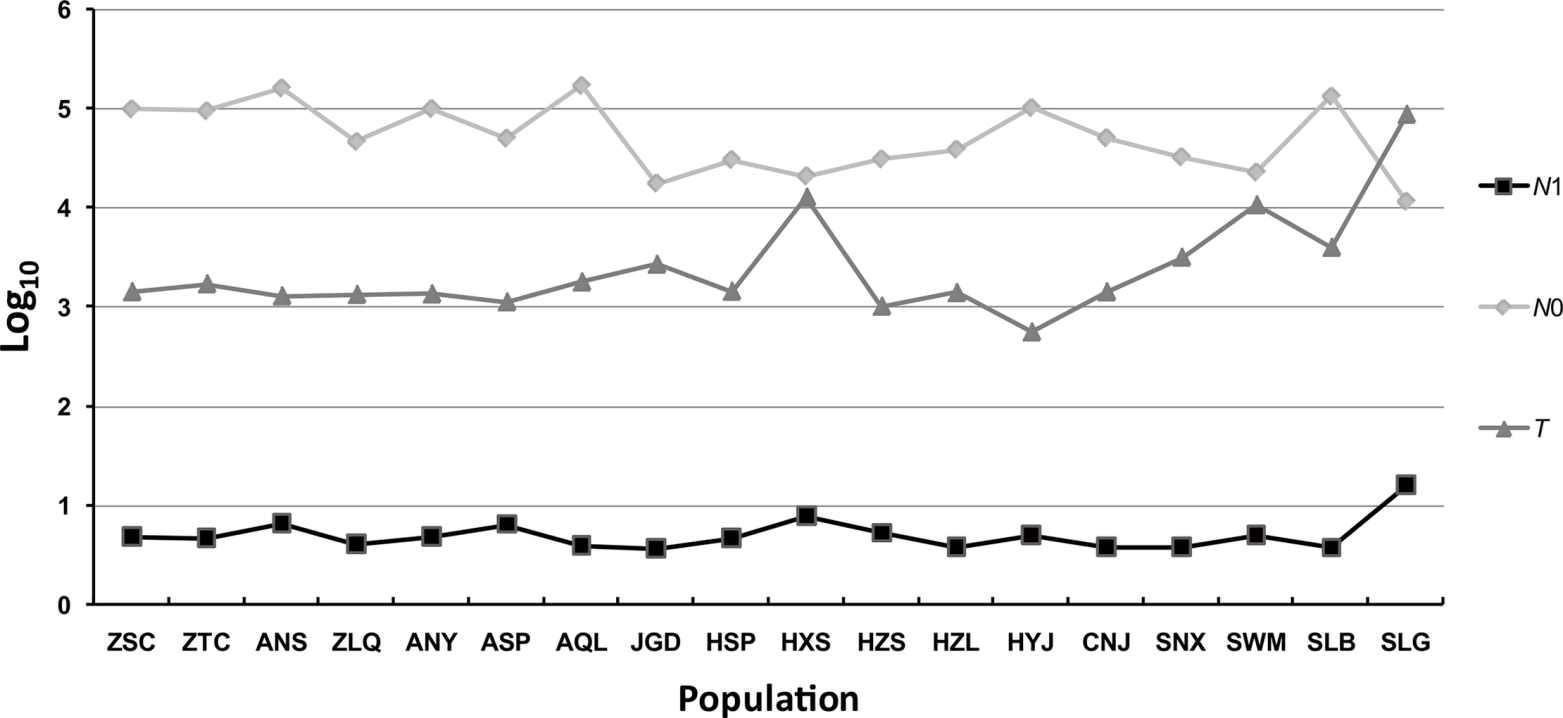
Effective population size and years since a change for 18 populations of *B*. *clarkeana*, based on the MSVAR analysis. *N*_1_, mean current population size (over loci); *N*_0_, mean ancestral population size; *T*, mean time (in years) since the population started to decline.

According to the MSVAR results, the mean effective population size of all populations of *B*. *clarkeana* has experienced a dramatic reduction (see Fig 5). The mean ancestral population size was larger in the eastern region than in the other regions. Population SLG exhibited the largest current population size, with all other populations presenting a similar size. Most of the populations started to decline approx. 1,000–10,000 yr ago, except for populations SLG (~ 100,000 yr ago) and HYJ (< 1,000 yr ago). In addition, based on integrating the results regarding recent changes in effective population size tested in BOTTLENECK, seven populations showed strong evidence of recent bottlenecks. The bottlenecks mainly occurred in the eastern region, and the effect was particularly notable among the 7 populations of Mt. Huang-Tianmu, where 5 populations suffered bottleneck effects (see S6 Table). Two populations (HSP and HZL) in the central region also exhibited evidence of bottlenecks, and no bottlenecks were detected in the western region populations (Mt. Nan and Mt. Qinling-Daba). A clear progressive decrease in population bottlenecks from eastern to western regions was observed.

## Discussion

### Population genetic diversity

The population genetic diversity of *B*. *clarkeana* (*H*_O_ = 0.06, *H*_E_ = 0.08) revealed through EST-SSR analysis was significantly lower than the mean diversity values for perennial plants (*H*_O_ = 0.63, *H*_E_ = 0.68) and cross-pollinated plants (*H*_O_ = 0.63, *H*_E_ = 0.65), based on SSR data from Nybom (2004) [62]. The cpDNA and ITS data revealed the similar results. Most of the populations examined in this study showed only a single fixed haplotype, and population genetic diversity values were lowest among the studied plants in these areas [4, 7–9, 15, 63, 64]. This phenomenon of low population genetic diversity is due to several factors. First, *B*. *clarkeana* is a facultatively outcrossing and highly self-compatible species, and these features were reflected in the significant inbreeding coefficient [18]. Second, a substantial number of the populations may have suffered from genetic bottlenecks. Excess heterozygosity and allelic deficiency can also lead to low population genetic diversity. In addition, the habitat restriction and small population sizes of this plant in its natural state are important factors leading to low genetic diversity. The short stature of *B*. *clarkeana* and its growth on the north side of rock outcrops (mostly limestone), requirement for scattered light and growth in the shadow of trees and shrubs are all habitat restrictions that might significantly reduce the dispersal capacity of windborne seeds and the frequency of pollination, which could lead to reproductive isolation of a population or across a region [65]. By means of orientational observations in the field, Zhang (2011) found that many populations of *B*. *clarkeana* exhibit a shortage of visiting pollinators as well as a reduced frequency [21]. Moreover, field investigation have revealed a small size for most populations, which usually consist of no more than 50 individuals, while a few populations only comprise several individuals. Small plant populations are usually subject to the highest genetic risk of inbreeding and genetic drift, which can alter the genetic diversity and fitness of populations [66]. All of these characteristics decrease genetic variation within populations of *B*. *clarkeana*.

### Genetic structure

The different genetic information and evolutionary rates between cpDNA and nuclear DNA (nDNA) [67, 68] and, thus, differences in molecular markers can result in different genetic structures [8, 12]. Accordingly, multiple molecular markers may complement each other, which would aid in understanding the genetic structure of species [69]. Based on the analysis of cpDNA, which is seed dispersed and maternally inherited, a significant barrier to seed flow was confirmed by the network and the phylogenetic tree, as we found that two adjacent regions showed haplotypes with distant relationships (Mt. Shennongjia-Wu and Mt. Zhangjiajie, Mt. Zhangjiajie and Mt. Nan). In comparison with maternal cpDNA, which exhibits biparental gene flow and a higher evolutionary rate, the nDNA EST-SSR and ITS data shared the characteristic of most populations being clustered in their regions in the generated network and phylogenetic trees. For example, the haplotype of Mt. Guan (C3) was located at a tip linked to Mt. Qinling-Daba (C7) in the cpDNA network, whereas the ribotype of Mt. Guan (R5) was located at a tip linked to Mt. Huang-Tianmu (R1). However, the PCoA and phylogenetic tree for the EST-SSR data showed a different structure from those for the cpDNA and ITS data, as the populations of Mt. Huang-Tianmu and Mt. Qinling-Daba did not exhibit a close relationship according to the EST-SSRs, which are located within expressed genes and may have undergone long-term adaptive evolution in response to the environment. The particular location in the genome as well as any transcribed products can affect the mutation patterns of SSRs [70, 71]. Due to the influence of the monsoonal wind system, precipitation and humidity on the Chinese mainland decrease longitudinally from east to west [72, 73]. These climatic variations lead to differences in gene expression in different areas; for example, the population dehydration tolerance of *B*. *clarkeana* has been confirmed to increase gradually from east to west [33]. Therefore, the expressed genes containing SSRs, which may affect the EST-SSR mutation rate, might have led to the different clustering results when comparing EST-SSR with cpDNA and ITS. Overall, synthesis of the results of detection of the three markers confirmed low genetic variation within a population or region, but high genetic variation between populations from different regions [66, 74–77]. As a result, the pairwise *F*_ST_ values based on the EST-SSR data were significantly higher (most greater than 0.5), and the differentiation indices *G*_ST_ and *N*_ST_ (cpDNA, 0.964, 0.995; ITS, 0.862, 0.931) were also significantly higher than the mean values (maternally inherited markers, *G*_ST_ = 0.655, *N*_ST_ = 0.69; biparental, *G*_ST_ = 0.163; *N*_ST_ = ~0.23) between plants [78].

### Glacial refugia and colonization

In this study, based on the TCS networks of cpDNA, haplotypes C2 of Mt. Huang-Tianmu and C7 of Mt. Qinling-Daba were both considered to be ancestral for *B*. *clarkeana*. In addition, high cpDNA haplotype and nucleotide diversity is a feature of refugia [5]. In this study, high haplotype and nucleotide diversities were only found in two populations: ZSC (Mt. Huang-Tianmu) and SNX (Mt. Qinling-Daba). These results indicate that Mt. Qinling-Daba and Mt. Huang-Tianmu represent potential refuge areas for this plant species, even though these two regions are located far apart and are near the southeastern and northwestern limits of the *B*. *clarkeana* distribution. The Mt. Huang-Tianmu and Mt. Qinling-Daba areas were previously shown to harbor refugia for other species during the LGM (approx. 24,000–18,000 yr BP) [5, 6, 63, 79–82].

For cpDNA, a significant and moderately strong correlation between genetic and geographic distance between the populations was revealed by Mantel tests (*r*_M_ = 0.54, *P* < 0.01), reflecting the 'isolation by distance' status of *B*. *clarkeana*. However, compared with other studies conducted in this area, the *r*_M_ value of *B*. *clarkeana* was even higher than that of the rare plant *Dysosma versipellis* (*r*_M_ = 0.44) [83] and was only lower than that of the relict *Tetracentron sinense* (*r*_M_ = 0.62) [9]; the *r*_M_ value for *B*. *clarkeana* was relatively higher. These findings indicate the occurrence of an extinction event in the Pleistocene [84]. Similar to many other plant species, the long species history of *B*. *clarkeana* enabled it to expand throughout the middle and lower reaches of the Yangtze River. The analyses of gene flow and effective population size variation also showed that the studied populations of *B*. *clarkeana* were historically large and widely distributed (significant historical gene flow and a large historical population size). However, extinction occurred due to climate change in the Pleistocene, and *B*. *clarkeana* was forced into two refugia (Mt. Huang-Tianmu and Mt. Qinling-Daba) by the end of Pleistocene [84–86]. According to previous studies, there is a general colonization route in this area from Mt. Huang-Tianmu to Mt. Shennongjia, passing through Mt. Dabie (see Fig 1A) [79, 87]. However, *B*. *clarkeana* is not currently found in Mt. Dabie, which may constitute evidence of extinction for this species. A lack of molecular variation within the regions was revealed through phylogeographic analysis (e.g., genetic variation mostly existed between regions, and no haplotypes were shared between regions), which also supports the fragmentation and extinction of *B*. *clarkeana* [5, 9, 84]. Because of its weak seed dispersal ability, *B*. *clarkeana* would not have been able to undergo long-distance expansion into suitable territories during interglacial periods, which may also have resulted in distant haplotype relationships in adjacent areas (Mt. Shennongjia-Wu and Mt. Zhangjiajie, Mt. Zhangjiajie and Mt. Nan). Therefore, no evidence that *B*. *clarkeana* had undergone a significant southward retreat during glacial periods and northward migration during interglacial periods was found. Similar to some other studies [9, 88–90], no clear long colonization route was identified for *B*. *clarkeana*, only local expansion (from Mt. Qinling-Daba, 121,457 yr BP) occurring under suitable climatic conditions during the interglacial period (approx. 125,000–70,000 yr BP).

### Human influence on effective population size reduction

According to the MSVAR results, the mean effective population size of all populations experienced a dramatic reduction, but the timing of the declines of most populations (approx. 1,000–10,000 yr ago) did not coincide with major climatic and landscape changes in this area during the Late Pleistocene (approx. 10,000–100,000 yr ago) [79]. Instead, environmental changes caused by human activities were inferred to be the main reason for the observed reduction [60, 79]. During the Yangshao Cultural period (approx. 5000–6000 yr BP), rapid agricultural growth caused serious forest degradation over a large area [91, 92], and such effects were especially prominent under the prosperous Hemudu Culture in the Yangtze River delta (5000–4500 yr BC) [93]. In this study, populations in the western region (Mt. Nan and Qinling-Daba) were not found to have been influenced by bottlenecks, although bottlenecks were detected in eastern and central regions, where intensive human activities are frequent and high-density populations exist, suggesting that bottlenecks were increased by human activities. Nevertheless, in dramatic contrast to the frequent occurrence of bottlenecks in the *B*. *clarkeana* populations in the lower reaches of the Yangtze River (Mt. Huang-Tianmu region), no bottlenecks were detected in *Kirengeshoma palmata* populations in this region in a previous study [60]. Although both herbs grow in wooded mountain environments, *K*. *palmata* lives in deep forests, whereas *B*. *clarkeana* grows on rocky outcrops and is more easily damaged during the clearing of land for agriculture.

## Conclusions

*B*. *clarkeana* is a resurrection herb species endemic to China. In this study, the genetic structure and evolutionary history of this species were investigated based on cpDNA, ITS and EST-SSR sequence data. Similar to other plant species of Gesneriaceae, this plant is restricted to rocky-slope habitats and exhibits weak seed and pollen dispersal abilities. The genetic diversity of *B*. *clarkeana* was found to be exceptionally low within each population and region. More suitable habitats for *B*. *clarkeana* were available historically, and the species was likely to be widely distributed. Because of changes in the environment and the influence of human activity, a few populations of *B*. *clarkeana* have become extinct, but Mt. Qinling-Daba and Mt. Huang-Tianmu have acted as refugia, conserving ancestral haplotypes of *B*. *clarkeana*. Habitat loss and fragmentation are becoming increasingly serious, and we should pay attention to the fact that a small population size is becoming the norm and that the genetic risk for this plant is increasing. Moreover, some populations have suffered genetic bottlenecks, particularly in the Mt. Huang-Tianmu (Yangtze River delta) area. These analyses indicate that climate and environmental change are important for species migration and preservation. We now have a better overall understanding of the influence of habitat restriction on *B*. *clarkeana* and for Gesneriaceae.

## Supporting information

S1 Table CpDNA sequence polymorphisms detected in three IGS (*psb*A-*trn*H, *rpl*20-*rps*12 and *trn*L-*trn*F) regions of *B*. *clarkeana*, identifying 8 haplotypes. (DOC)

S2 Table Genetic diversity and haplotype composition of the surveyed *B*. *clarkeana* regions, based on cpDNA (*psb*A-*trn*H, *rps*12-*rpl*20, *trn*L-*trn*F), ITS and EST-SSR data. (DOC)

S3 Table NrDNA sequence polymorphisms detected in the ITS region of *B*. *clarkeana*, Identifying 14 haplotypes. (DOC)

S4 Table Results of analysis of molecular variation of ITS sequences from different groups of *B*. *clarkeana*. (DOC)

S5 Table Mean historical migration rates among populations of *B*. *clarkeana*. (DOC)

S6 Table The result of bottleneck testing in 18 populations of *B*. *clarkeana*. (DOCX)

S1 Fig Distribution curve of pairwise nucleotide differences in cpDNA sequence data in the Mt. Qinling-Daba populations of *B*. *clarkeana*. (DOCX)
