# Supplementary material for: Molecular phylogeography of East Asian Boea clarkeana (Gesneriaceae) in relation to habitat restriction
Source: PLoS One. 2018 Jul 3;13(7):e0199780. doi: 10.1371/journal.pone.0199780 (PMC6029794; doi:10.1371/journal.pone.0199780)
Supplement: S1 Table — (DOC) [file pone.0199780.s001.doc]

**S1 Table. CpDNA sequence polymorphisms detected in three IGS (*psb*A-*trn*H, *rpl*20-*rps*12 and *trn*L-*trn*F) regions of *B. clarkeana*, identifying 8 haplotypes**

| **Nucleotide**  **position** | ***psb*A-*trn*H** | | | | | | | | | | | | | | | | | | | | | |  | ***rpl*20-*rps*12** | | | |  | ***trn*L-*trn*F** | | | | | | | | |
| --- | --- | --- | --- | --- | --- | --- | --- | --- | --- | --- | --- | --- | --- | --- | --- | --- | --- | --- | --- | --- | --- | --- | --- | --- | --- | --- | --- | --- | --- | --- | --- | --- | --- | --- | --- | --- | --- |
|  |  |  |  |  |  |  |  |  |  |  |  |  |  |  |  |  |  |  |  |  |  |  |  |  |  |  |  |  | 1 | 1 | 1 | 1 | 1 | 1 | 1 | 1 | 1 |
|  | 1 | 1 | 1 | 1 | 1 | 1 | 1 | 1 | 1 | 1 | 1 | 1 | 1 | 1 | 1 | 2 | 2 | 2 | 2 | 2 | 2 | 2 |  | 5 | 7 | 9 | 9 |  | 1 | 2 | 3 | 3 | 3 | 5 | 6 | 6 | 6 |
|  | 1 | 2 | 2 | 2 | 2 | 2 | 2 | 3 | 3 | 3 | 3 | 3 | 4 | 4 | 4 | 3 | 3 | 3 | 5 | 6 | 6 | 7 |  | 1 | 1 | 8 | 8 |  | 5 | 7 | 0 | 3 | 4 | 8 | 6 | 7 | 8 |
|  | 9 | 1 | 2 | 3 | 6 | 7 | 9 | 1 | 3 | 5 | 6 | 9 | 0 | 1 | 3 | 0 | 3 | 8 | 0 | 1 | 3 | 2 |  | 0 | 1 | 6 | 8 |  | 4 | 5 | 5 | 3 | 0 | 3 | 5 | 1 | 3 |
| H1 | A | T | A | T | C | T | G | T | T | C | C | G | A | G | G | T | A | T | A | A | A | A |  | C | C | A | C |  | T | C | C | C | G | A | C | A | A |
| H2 | . | . | . | . | . | . | . | . | . | . | . | . | . | . | . | . | . | . | . | . | . | . |  | . | . | . | . |  | . | . | . | A | . | . | . | . | . |
| H3 | . | . | . | . | . | . | . | . | . | . | . | . | . | . | . | G | . | G | . | . | . | . |  | . | . | . | . |  | . | . | . | A | . | G | . | . | . |
| H4 | . | . | . | . | . | . | . | . | . | . | . | . | . | . | . | G | C | . | C | . | . | . |  | T | . | C | T |  | C | . | . | A | . | . | . | . | . |
| H5 | . | . | . | . | . | . | . | . | . | . | . | . | . | A | . | G | . | . | . | . | . | T |  | . | . | . | . |  | . | A | T | A | . | G | . | . | . |
| H6 | C | C | T | C | G | G | A | A | C | A | G | A | T | A | T | . | . | G | . | T | T | . |  | T | . | . | . |  | . | . | . | A | A | . | T | T | G |
| H7 | . | . | . | . | . | . | . | . | . | . | . | . | . | . | . | G | . | . | . | . | . | . |  | . | . | . | . |  | . | . | . | A | . | G | . | . | . |
| H8 | . | . | . | . | . | . | . | . | . | . | . | . | . | . | . | G | . | . | . | . | . | . |  | . | T | . | . |  | . | . | . | A | . | G | . | . | . |
