# Supplementary material for: Molecular phylogeography of East Asian Boea clarkeana (Gesneriaceae) in relation to habitat restriction
Source: PLoS One. 2018 Jul 3;13(7):e0199780. doi: 10.1371/journal.pone.0199780 (PMC6029794; doi:10.1371/journal.pone.0199780)
Supplement: S2 Table — (DOC) [file pone.0199780.s002.doc]

**S2 Table. Genetic diversity and haplotype composition of the surveyed *B. clarkeana* regions, based on cpDNA (*psb*A-*trn*H, *rps*12-*rpl*20, *trn*L-*trn*F), ITS and EST-SSR data**

| **Region^a^** | **Size** | **EST-SSR^b^** | | | | | **cpDNA^c^** | | | **ITS** | | |
| --- | --- | --- | --- | --- | --- | --- | --- | --- | --- | --- | --- | --- |
|  |  | ***N*_A_** | **PIC** | ***H*_O_** | ***H*_E_** | ***F*_IS_** | **Haplotype** | ***h*** | **π×10^3^** | **Haplotype** | ***h*** | **π×10^3^** |
| Eastern | 181 | 1.392 | 0.076 | 0.068 | 0.091 | 0.274 | C1-3 | 0.02 | 0.01 | R1-5 | 0.07 | 0.13 |
| Central | 106 | 1.468 | 0.1 | 0.096 | 0.116 | 0.157 | C4,C5 | 0 | 0 | R6-11 | 0.25 | 1.01 |
| Southwest | 24 | 1.333 | 0.037 | 0.014 | 0.043 | 0.688 | C6 | 0 | 0 | R12 | 0 | 0 |
| Northwest | 83 | 1.08 | 0.013 | 0.014 | 0.015 | 0.048 | C7,C8 | 0.10 | 0.06 | R13,14 | 0.13 | 0.41 |

*Notes*:^a^: Eastern, Huang, Tianmu and Guan Mts.; Central, Wu, Shennongjia and Zhangjiajie Mts.; Southwest, N, Nan Mts.; Northwest, Qinling and Daba Mts.. ^b^, *N*_A_, number of alleles per locus across all populations; PIC, polymorphic information content; *H*_O_, observed heterozygosity (mean value); *H*_E_, expected heterozygosity (mean value); *F*_IS_, inbreeding coefficient. ^c^, *h* haplotype diversity; *π*, nucleotide diversity.
