# Supplementary material for: Molecular phylogeography of East Asian Boea clarkeana (Gesneriaceae) in relation to habitat restriction
Source: PLoS One. 2018 Jul 3;13(7):e0199780. doi: 10.1371/journal.pone.0199780 (PMC6029794; doi:10.1371/journal.pone.0199780)
Supplement: S3 Table — (DOC) [file pone.0199780.s003.doc]

**S3 Table. NrDNA sequence polymorphisms detected in the ITS region of *B. clarkeana*, identifying 14 haplotyp**es

| **Nucleotideposition** |  |  | 1 | 1 | 1 | 1 | 1 | 1 | 1 | 2 | 2 | 4 | 4 | 5 | 5 | 5 | 5 | 6 | 6 | 6 | 6 | 6 |
| --- | --- | --- | --- | --- | --- | --- | --- | --- | --- | --- | --- | --- | --- | --- | --- | --- | --- | --- | --- | --- | --- | --- |
|  | 4 | 6 | 2 | 4 | 7 | 7 | 8 | 8 | 8 | 5 | 5 | 0 | 9 | 1 | 4 | 5 | 6 | 1 | 3 | 4 | 4 | 6 |
|  | 3 | 7 | 8 | 3 | 3 | 5 | 5 | 6 | 9 | 1 | 2 | 1 | 1 | 4 | 9 | 8 | 5 | 8 | 1 | 7 | 8 | 0 |
| R1 | A | G | T | T | A | T | T | C | T | A | A | T | A | T | C | T | A | G | A | A | G | T |
| R2 | . | . | . | . | . | . | . | . | . | T | . | . | . | . | . | . | . | . | . | . | . | . |
| R3 | . | . | . | . | G | . | . | . | . | T | . | . | . | . | . | . | . | . | . | . | . | . |
| R4 | . | . | C | . | . | . | . | . | . | T | . | . | . | . | . | . | . | . | . | . | . | . |
| R5 | G | . | . | . | . | . | . | . | . | . | . | . | . | . | . | . | . | . | . | . | . | . |
| R6 | . | . | . | . | . | . | A | A | . | . | . | . | . | . | A | C | . | T | . | T | A | C |
| R7 | . | . | . | . | . | . | A | A | . | . | . | . | . | . | A | . | . | T | . | T | A | C |
| R8 | . | . | . | . | . | . | A | A | . | . | . | . | . | . | A | . | . | . | . | T | A | C |
| R9 | . | . | . | . | . | . | . | . | . | . | . | . | G | . | . | . | G | . | G | . | . | . |
| R10 | . | . | . | C | . | . | . | . | . | . | G | . | G | . | . | . | . | . | . | . | . | . |
| R11 | . | . | . | . | . | . | . | . | C | . | . | . | . | . | . | . | . | . | . | . | . | . |
| R12 | . | . | . | C | . | . | . | . | . | . | . | C | G | C | . | . | . | . | . | . | A | . |
| R13 | . | . | A | . | . | C | . | . | . | . | . | . | . | . | . | . | . | . | . | . | . | . |
| R14 | . | T | . | . | . | C | . | . | . | . | . | . | . | . | . | . | . | . | . | . | . | . |
