# Supplementary material for: Molecular phylogeography of East Asian Boea clarkeana (Gesneriaceae) in relation to habitat restriction
Source: PLoS One. 2018 Jul 3;13(7):e0199780. doi: 10.1371/journal.pone.0199780 (PMC6029794; doi:10.1371/journal.pone.0199780)
Supplement: S4 Table — (DOC) [file pone.0199780.s004.doc]

***S4 Table. Results of analysis of molecular variation of ITS sequences from different groups of B. clarkeana***

| **Groups^a^** | **Percentage of variation** | | | | ***G*_ST_(se)** | ***N*_ST_(se)** |
| --- | --- | --- | --- | --- | --- | --- |
|  | Among  groups | Among populations | Within  populations | *Φ*_ST_ |  |  |
| H-T | 46.07 | 45.61 | 8.31 | 0.917 | 0.903 (0.066) | 0.926 (0.050) |
| H-T-G | 14.93 | 77.59 | 7.48 | 0.925 | 0.921(0.057) | 0.943(0.042) |
| Z-W-S | 80.33 | 11.56 | 8.11 | 0.919 | 0.69(0.173) | 0.893(0.046) |
| WL(Z, N) | 33.05 | 52.16 | 14.79 | 0.852 | 0.810(0.192) | 0.849(0.177) |
| W-S-Q-D | 94.41 | 2.08 | 3.51 | 0.965 | 0.747(0.144) | 0.956(0.027) |
| Q-D | -2.11 | 38.33 | 63.77 | 0.362 | 0.364 (NC) | 0.364 (NC) |
| Total | 75.08 | 19.4 | 5.52 | 0.945 | 0.862(0.059) | 0.931(0.037) |

*Note*:^a^: H, Huang Mts.; T,Tianmu Mts.; G, Guan Mts.; W, Mt. Wu; S, Shennongjia Mts.; Z, Mt. Zhangjiajie; N, Nan Mts.; WL, Mt.Wuling; Q, Mt.Qinling; D, Daba Mts..
