# Supplementary material for: Molecular phylogeography of East Asian Boea clarkeana (Gesneriaceae) in relation to habitat restriction
Source: PLoS One. 2018 Jul 3;13(7):e0199780. doi: 10.1371/journal.pone.0199780 (PMC6029794; doi:10.1371/journal.pone.0199780)
Supplement: S5 Table — (DOC) [file pone.0199780.s005.doc]

**S5 Table. Mean historical migration rates among populations of *B. clarkeana***

| **Population** | **ZSC** | **ZTC** | **ZLQ** | **ANS** | **ANY** | **ASP** | **AQL** | **JGD** | **HSP** | **HXS** | **HZS** | **HZL** | **HYJ** | **CNJ** | **SNX** | **SWM** | **SLB** | **SLG** |
| --- | --- | --- | --- | --- | --- | --- | --- | --- | --- | --- | --- | --- | --- | --- | --- | --- | --- | --- |
| **ZSC** | 0 | 0.122 | 0.138 | 0.06 | 0.14 | 0.192 | 0.053 | 0.068 | 0.106 | 0.082 | 0.1 | 0.067 | 0.11 | 0.131 | 0.051 | 0.072 | 0.063 | 0.104 |
| **ZTC** | 0.138 | 0 | 0.116 | 0.199 | 0.142 | 0.075 | 0.171 | 0.14 | 0.104 | 0.174 | **0.217** | 0.014 | 0.117 | 0.059 | 0.072 | 0.121 | 0.119 | 0.079 |
| **ZLQ** | **0.213** | 0.118 | 0 | 0.135 | **0.211** | 0.073 | 0.065 | 0.079 | 0.06 | 0.094 | 0.129 | 0.123 | 0.102 | 0.115 | 0.104 | 0.077 | 0.082 | 0.105 |
| **ANS** | 0.168 | 0.179 | 0.14 | 0 | 0.086 | 0.186 | 0.149 | 0.185 | 0.021 | 0.197 | 0.154 | 0.078 | 0.148 | 0.194 | 0.049 | 0.07 | 0.096 | 0.079 |
| **ANY** | 0.179 | 0.187 | 0.07 | 0.101 | 0 | 0.099 | 0.067 | 0.116 | 0.1 | 0.156 | 0.12 | **0.207** | 0.103 | 0.076 | 0.143 | 0.079 | 0.119 | 0.151 |
| **ASP** | 0.181 | 0.142 | 0.092 | 0.143 | 0.17 | 0 | 0.164 | 0.162 | 0.139 | 0.168 | 0.079 | 0.121 | 0.15 | 0.154 | 0.182 | 0.043 | 0.103 | 0.143 |
| **AQL** | 0.163 | **0.242** | 0.158 | 0.179 | 0.086 | **0.228** | 0 | 0.087 | 0.119 | 0.183 | 0.08 | 0.14 | 0.148 | 0.095 | 0.162 | 0.097 | 0.127 | 0.187 |
| **JGD** | 0.161 | 0.119 | 0.136 | 0.026 | 0.147 | 0.149 | 0.169 | 0 | 0.083 | 0.092 | 0.149 | 0.135 | 0.127 | 0.132 | 0.1 | 0.16 | 0.146 | 0.106 |
| **HSP** | 0.14 | 0.045 | 0.053 | 0.113 | 0.036 | 0.142 | 0.106 | 0.064 | 0 | **0.202** | 0.178 | 0.14 | 0.101 | 0.163 | 0.113 | 0.122 | 0.103 | 0.159 |
| **HXS** | 0.099 | 0.128 | 0.174 | 0.178 | 0.114 | 0.107 | 0.175 | 0.127 | 0.162 | 0 | 0.159 | 0.09 | 0.114 | 0.118 | 0.028 | 0.104 | 0.143 | 0.122 |
| **HZS** | 0.054 | 0.099 | 0.098 | 0.131 | 0.029 | 0.082 | 0.033 | 0.072 | 0.086 | 0.129 | 0 | 0.106 | 0.046 | 0.061 | 0.119 | 0.178 | 0.077 | 0.043 |
| **HZL** | 0.026 | 0.161 | 0.09 | 0.019 | 0.088 | 0.124 | 0.12 | 0.052 | 0.111 | 0.097 | 0.118 | 0 | 0.193 | **0.204** | 0.175 | 0.074 | 0.073 | 0.101 |
| **HYJ** | 0.08 | 0.096 | 0.124 | 0.071 | 0.022 | 0.108 | 0.096 | 0.117 | 0.177 | 0.047 | 0.058 | **0.204** | 0 | 0.126 | 0.07 | 0.159 | 0.062 | 0.148 |
| **CNJ** | 0.043 | 0.028 | 0.143 | 0.033 | 0.092 | 0.02 | 0.036 | 0.012 | 0.054 | 0.064 | 0.049 | 0.028 | 0.068 | 0 | 0.056 | 0.008 | 0.027 | 0.065 |
| **SNX** | 0.104 | 0.082 | 0.159 | 0.112 | 0.161 | 0.137 | 0.066 | 0.099 | 0.086 | 0.106 | 0.106 | 0.193 | 0.067 | 0.084 | 0 | 0.169 | 0.158 | 0.138 |
| **SWM** | 0.036 | 0.086 | 0.066 | 0.141 | 0.1 | 0.08 | 0.06 | 0.149 | 0.145 | 0.159 | 0.1419 | **0.206** | 0.06 | 0.127 | 0.152 | 0 | 0.186 | 0.107 |
| **SLB** | 0.092 | 0.048 | 0.122 | 0.071 | 0.075 | 0.094 | 0.076 | 0.06 | 0.121 | 0.071 | 0.128 | 0.121 | 0.076 | 0.083 | 0.17 | 0.195 | 0 | **0.213** |
| **SLG** | 0.088 | 0.113 | 0.058 | 0.026 | 0.081 | 0.037 | 0.065 | 0.06 | 0.096 | 0.096 | 0.114 | 0.199 | 0.108 | 0.115 | **0.215** | 0.194 | **0.248** | 0 |

*Note:* The direction of migration (gene flow) is from ordinate populations to abscissa populations. Values of *m*h greater than 0.2 are in bold.
