# Supplementary material for: Molecular phylogeography of East Asian Boea clarkeana (Gesneriaceae) in relation to habitat restriction
Source: PLoS One. 2018 Jul 3;13(7):e0199780. doi: 10.1371/journal.pone.0199780 (PMC6029794; doi:10.1371/journal.pone.0199780)
Supplement: S6 Table — (DOCX) [file pone.0199780.s006.docx]

**S6 Table. The result of BOTTLENECK testing in 18 populations of *B. clarkeana***

| **Population** | **IAM** | **TPM** | **SMM** | **Mode-shift** |
| --- | --- | --- | --- | --- |
| ZSC | 0.813 | 1.000 | 0.813 | L-shaped |
| **ZTC** | 0.188 | 0.313 | 0.313 | **shifted mode** |
| ZLQ | 0.188 | 0.313 | 0.313 | L-shaped |
| **ANS** | **0.023** | **0.016** | **0.008** | L-shaped |
| **ANY** | 0.094 | 0.094 | 0.625 | **shifted mode** |
| **ACT** | 0.938 | 0.688 | 0.375 | **shifted mode** |
| **AQL** | 0.188 | 0.313 | 0.313 | **shifted mode** |
| JGD | 0.250 | 0.250 | 0.250 | L-shaped |
| **HSP** | 0.188 | 0.313 | 0.875 | **shifted mode** |
| HXS | 0.500 | 0.500 | 0.500 | L-shaped |
| HZS | 0.563 | 0.563 | 0.563 | L-shaped |
| **HZL** | 0.125 | 0.313 | 0.875 | **shifted mode** |
| HYJ | 0.019 | 0.131 | 0.322 | L-shaped |
| CNJ | 0.125 | 0.125 | 0.125 | L-shaped |
| SNX | 0.500 | 0.500 | 0.500 | L-shaped |
| SWM | 0.500 | 0.500 | 0.500 | L-shaped |
| SLB | 0.500 | 0.500 | 0.500 | L-shaped |
| SLG | 1.000 | 1.000 | 1.000 | L-shaped |

*Note*: IAM, infinite alleles model; TPM, two-phase mutation model; SSM, stepwise mutation model. The notable bottleneck information (code and test value) is in bold.
