# Supplementary material for: Molecular phylogeography of East Asian Boea clarkeana (Gesneriaceae) in relation to habitat restriction
Source: PLoS One. 2018 Jul 3;13(7):e0199780. doi: 10.1371/journal.pone.0199780 (PMC6029794; doi:10.1371/journal.pone.0199780)
Supplement: S1 Fig — (DOCX) [file pone.0199780.s007.docx]

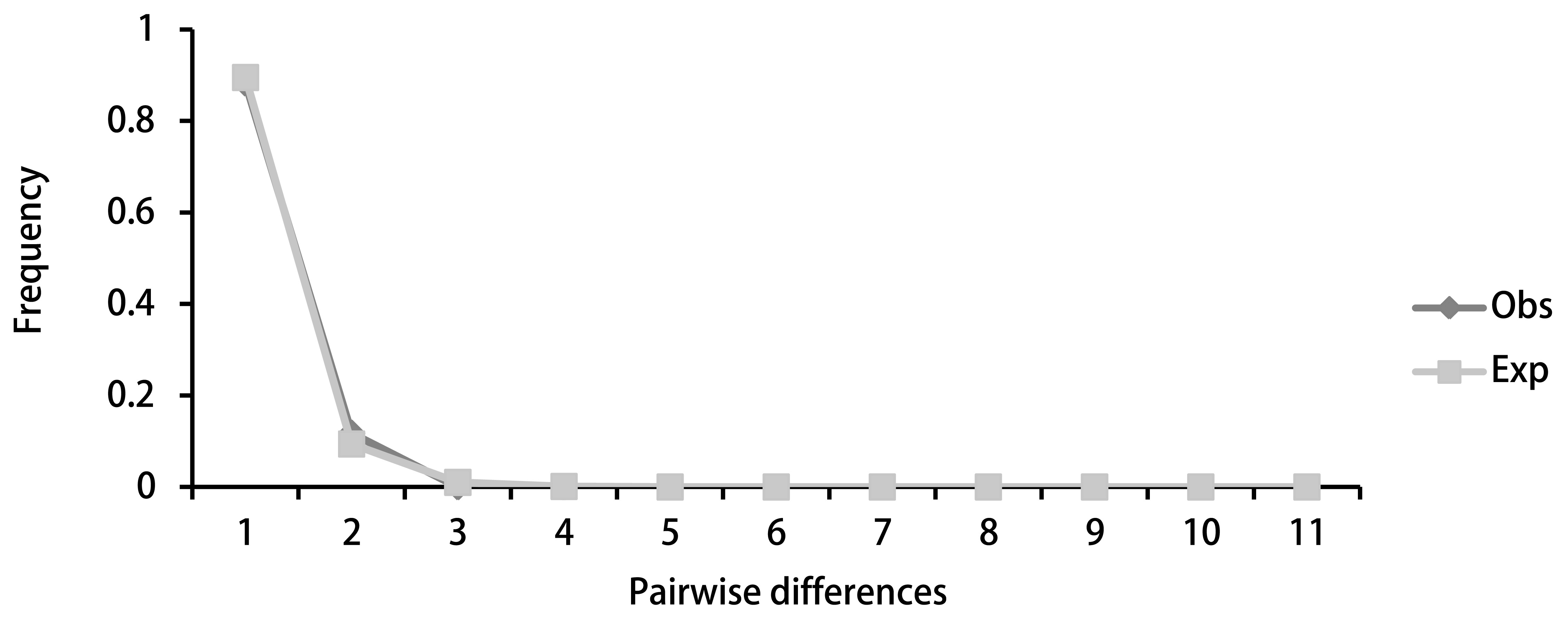


**S1 Fig. Distribution curve of pairwise nucleotide differences in cpDNA sequence data in the Mt. Qinling-Daba populations of *B. clarkeana*.** The dark gray line represents the observed frequency, and the light gray line shows the expected value of population expansion.
